# Economic Impact of Temperature Control during Food Transportation—A COVID-19 Perspective

**DOI:** 10.3390/foods11030467

**Published:** 2022-02-04

**Authors:** Eulalia Skawińska, Romuald I. Zalewski

**Affiliations:** Faculty of Management and Economics, University of Zielona Góra, 65-417 Zielona Góra, Poland; r.zalewski@wez.uz.zgora.pl

**Keywords:** economic food availability, food waste and losses, temperature abuse, SPC charts, economic loss, FCC, COVID-19

## Abstract

Temperature fluctuation and abuse in the food cold chain (FCC) is becoming an increasingly crucial factor in the process of food production and for the logistic business, especially in COVID-19 pandemic. The quality of perishable food products depends largely on accurate transport and maintenance temperature. The evidence for temperature-related food waste and loss is extensive. The research problem is thus: how to decrease and control food losses caused by temperature abuse in the FCC and restrictions due to the COVID-19 pandemic. The primary objective is to propose a framework for real-time temperature measurement protocols supported by passive RFID, IoT and Statistical Process Control (SPC) charts. This method allows not only the signaling of temperature abuse alerts but, in addition to hitherto methods, investigation and mitigation of the causes of process instability of individual FCC links in the future. The secondary objective is to delineate the necessary data sources and ways of their collection and utilization in order to decrease food losses and waste via process stabilization of temperature in transport and storage. As contribution to current literature and practice, we offer an in-depth analysis of threats in the FCC in food transport and storage infrastructure and a solution supplemented by SPC charts and tested in controlled experiments that is practicable from economic and technical standpoints.

## 1. Introduction

Extensive data suggest the necessity to reduce the amount of food waste and losses (FWL) along the entire food supply chain, including distribution. For example, total FWL per capita by consumers, in production and in retail, is almost 300 kg/year in highly developed countries and approximately half of this amount in underdeveloped countries [1,2,3]. Approximately 22 percent of food in the developed countries is discarded in consumer homes [4,5]. Ishangulyyev et al. (2019) [6] wrote that monetary value of FLW was estimated at about 936 billion USD. The demand for food worldwide will have increased by approximately 150 percent by 2050 (FAO, 2013) [7]. The European Union, facing and trying to solve similar problems, conducted research to better understand the drivers behind these problems and to identify potential solutions. In 2012, the European Commission (2012) [8] adopted a resolution to reduce food waste by 50 percent by 2020 and designated the year 2014 as the “European year against food waste” (European Commission, 2014) [9]. Unfortunately, a recent report (IPES, 2019) [10] identifies “…that 20% of the food produced in the European Union is wasted with the cost of 143 billion EUR annually”. All this has decreased economic food availability. According to the UNEP Food Waste Index 2021 (UNEP, 2021) [11], around 931 million tonnes of food waste were generated in 2019—61% of which came from households, 26% from food service and 13% from retail.

The problem of food waste and losses is also a vital issue regarding the present and projected effects of climate change on food production and nutrition security. In August 2019, the Intergovernmental Panel on Climate Change (IPCC) adopted a special report on the impact of climate change on the terrestrial ecosystem [12]. Food and nutrition security (i.e., availability, access, utilization, stability), food systems, farming systems, food–energy–water relations, the role of desertification and land degradation now and in future were highlighted and linked to climate change, globally and regionally. The report presents impacts of dramatic climate change on food and nutrition security, including food production, prices and livelihoods. It warns the global society that our civilization will shortly be confronted with many other challenges. From our point of view, two of these challenges are crucial to food security: rapid increase in population numbers, especially in countries already facing problems in food security, and overproduction and waste of food resources in highly developed countries. According to the United Nations, the global population will have increased from 6.3 billion now to approximately 9.5 billion people in 2050 and will grow to 11 billion by 2100. Another problem is that these projections refer mostly to underdeveloped countries, where the rate of population growth is already faster than food production, which further raises the prevalence of undernourishment (GFSI, 2020) [13]. In developed countries, we see food overproduction and FW as a result of poor surplus management (Capone et al. 2014) [14]. In these countries, the excess and the poor structure of consumption are a bigger problem than malnutrition, the latter affecting only 2.5% of the total population (FAO, 2017) [15]. However, irregularities in food consumption in rich countries also bare harmful effects for health and negative economic consequences (e.g., reduced labor productivity, increased costs of introducing new formal institutions) and thus reduce the quality and expectancy of life, which constitutes a social cost. The COVID-19 pandemic, especially the initial period, caused a demand and supply shock in many countries [16,17]. Among others, the role of logistics and climatic conditions and especially temperature has become clearer. From autumn 2019, supply chains have become a strategic factor in maintaining food security in many countries. Reports from international organizations provide information about the following changes in the food market: the increase in demand for organic and fresh food; the increase in malnutrition in underdeveloped countries; and the decrease in quality due to adulteration, counterfeiting, improper transport and storage, which leads to waste and wastage.

According to Berners-Lee et al. [18], the global food production will be sufficient to meet the nutritional needs of the human population in 2050 only if a radical societal adaptation takes place. Underdeveloped countries and regions, in which food and water shortage is already a problem, will require special attention in the future. Simultaneously, the opposite problem of food overproduction and/or overconsumption, illness from malnutrition and food waste in highly developed countries needs to be solved, as well. Recently, some research results have been published addressing another aspect of the problem: production of food is resource-intensive with a broad use of resources (e.g., water and energy consumption), and impact on the environment (greenhouse gas emission) [6,19]. Hence, it seems imperative for humanity to save and treasure our food, water and energy resources using all possible means, with an effective FCC (Godfray et al. 2010) [20].

Although, as the abovementioned data clearly indicate, the issue of food waste and loss is extensive and dire, there are still little systematic research and data to be found referring to the share of the cold chain inefficiency in this phenomenon. However, recent studies by RASSF suggest indirectly that temperature abuse in the FFC is a common incident [21]. Burnson reports that in the FCC, “abuses above or below the optimal product specific temperature range occur frequently [22]. This fact significantly increases food waste and jeopardizes safety”. It is noteworthy that this situation exists despite a revolutionary progress of temperature measurement and recording. In this paper, we analyzed secondary sources concerning food quality and economic losses in the food production chain caused by inadequate storage and transport temperature, especially in the cold part of the food chain. Based on literature review, we present an overview of the current technical solutions for temperature monitoring during transportation. However, there is a research gap in the literature and praxis. If during transport the temperature limit is exceeded, typically an alarm signal is generated and recorded, yet, this information is not further analyzed by managers for exploration of causes. As a solution, we offer a background for the time–temperature measurement protocols supported by passive RFID, IoT and SPC charts. This method allows not only signaling temperature abuse alerts, but unlike other methods currently in use, it also enables managers and transport operators to investigate and mitigate the causes of temperature instability of individual FCC links. The aim of this paper is to analyze the reasons for temperature abuse in the food transport chain and to develop a viable technical solution for better temperature management.

The literature on the subject offers numerous textbooks and references regarding the impact of temperature on food quality and safety. However, these are usually either general in nature or refer to fragments of the food chain only. Thus, our primary objective is to propose an application of SPC methods and tools in the temperature management of individual CC (cold chain) links applicable throughout the entire FCC. The supplementary objective is to delineate the necessary data sources and ways of their collection and utilization in order to decrease food losses and waste via stabilization of temperature in transport and storage process.

We found that typically only a part of the potential possibilities and information gathered in the temperature management system is applied to stabilize temperature in the FCC. The need to invest in the dissemination of temperature measurement technologies and the role of human capital as well as the importance of informal institutions, i.e., social capital, were pointed out. Recently announced integration with GPS expands the capabilities of temperature monitoring devices even more, thus offering additional data. We offer a framework for the time–temperature measurement protocols supported by passive RFID, IoT and SPC charts, which allows not only signaling of temperature abuse alerts but also enables investigation and mitigation of causes for process instability in individual FCC links. The results of our research may be useful for activities related to food traceability, changes in the European law, and encouraging the use of extensive methods of temperature measurements and SPC tools of transported food.

Harmonious and judicious application of appropriate statistical methods combined with the use of modern technologies for measuring temperature (and other parameters) supported by the Internet and employees with high social capital attributes such as integrity, faith and dignity, competence, responsibility and honesty, values which are closely related to motivational instruments for effective work, will be the right way to go. Only in this way will we be able reducing risks to food waste. All employees in the FCC must know and understand the scientific/technical basis of their operations.

Key results of our research are listed below:

1. A gap in the literature has been identified in the study of reasons for food losses in transport versus processing resulting from temperature overshoot.
2. A cause and effect analysis was performed in the form of management interviews with CEOs of large shipping companies, food wholesalers, retail chains, IoT specialists, NFC Data Loggers, manufacturers and researchers.
3. Five groups of factors with significant impact on food temperature during transportation were generated.
4. It was shown that food carriers using modern temperature recording methods at most react to an alarm signal, but they do not analyze the causes.
5. The use of statistical methods and SPC charts (averages, ranges and regressions) to study the causes of disturbances was proposed.

The paper is divided into five parts. The first offers an outline of the conducted research. The second part explains the experimental methods. The third—theoretical—explains the key terminology used in the paper: food safety and food security, FWL and the FCC, which are linked directly to microbiological spoilage and risk. It further presents the tools and technology currently implemented for temperature measurement based on the RFID and Internet of Things (IoT) and current limitations. The fourth part describes the application of SPC tools to increase the effectiveness of temperature management in the FCC and to reduce FWL throughout the entire food cold chain. It offers examples of SPC augmentation for typical temperature abuse cases and suggestions for the necessary data sources and ways of their collection and utilization. Finally, the fifth part concludes our key findings in the light of the presented context of FWL.

## 2. Materials and Methods

We conducted our research in two steps. First, we completed a comprehensive systematic literature analysis in order to explore research gaps and to inform our experimental research. In the latter, we developed and tested temperature control methodology, which allows us not only to register temperature abuse but also to explore its causes.

### 2.1. Systematic Literature Review

The method of systematic literature review enables a formalized and objectified synthesis of the scientific achievements to date [23]. It allows the identification of explored areas and makes it easier to see the unexplored [24,25].

The systematic literature review applied for the purpose of our research covered three stages [24,25,26]: (1) determining the purpose of the study and the basic literature, (2) selecting suitable publications and developing a database and (3) analyzing the content and verifying the applicability of the results obtained for further research. In the first phase, three databases were used: EBSCO, ProQuest and WoS, all of which are recommended for a systematic review of literature in context of social and economic sciences [27]. The following key phrases were used in the search: food waste and losses, food waste accounting methods, economic food availability, food cold chain, temperature abuse in food cold chain, temperature changes in transport, methods of temperature measurement, data transmission and visualization, application of SPC and food transport and statistical process control charts. Only scientific articles published in leading journals, articles in conference proceedings, chapters in monographs and reports published by international organizations were included in the pool of publications. The majority of the selected material was found in references devoted to food science and control, computer engineering, transport, quality management, SPC and economics. In the next step, we grouped the selected references into four main topics linked to the subject of our study: food waste and losses, food cold chain, temperature monitoring methods in transport and statistical process control. We observed that the literature on the use of control charts directly in the field of temperature changes in food transport was scarce.

### 2.2. Experimental Part

In our experiments, we used NFC Data Loggers delivered by Blulog. These loggers possess the following technical performance: the temperature range from −30 °C to +70 °C, a time interval from 2 s to 2 h, the scale of 0.1 °C, precision of 0.2–0.4 °C and the memory capacity of up to 48,000 measurements. The data gathered by the sensors are sent in an encrypted form via Internet to a hub, and the measurement results are presented in numerical and graphical form on the screen of a smartphone or a tablet. The application was programmed to send e-mail or SMS notifications in certain situations.

In the cargo room of a car (a refrigerated space with a load of 1 ton), food of one type was loaded in openwork plastic containers in five staples. Temperature sensors were distributed in the following way: two were placed between layers 1 and 2 diagonally square from right to left; the other two were placed between the fourth and fifth layer in left-to-right order following the Taguchi model [28]. Measurements of time, temperature and other parameters were used to develop plots and Statistical Control Charts (SPC charts) which were presented and analyzed in the following sections of the review. We also conducted managerial interviews with CEOs of large food trade and transport chains, medium retailers organized in franchising networks in Poland, as well as with quality managers, scholars from food sciences and specialists in IoT, resulting in an analysis of problems with temperature stability during transport of food (own unpublished case study). We applied the cause-and-effect diagram to organize our findings (Ishikawa’s method). This diagram “organizes the free-flowing of ideas into a logical pattern. With a little practice the diagram can be used very effectively when the group seeks to establish the cause of an effect which may be either a problem or a desirable effect” (Oakland and Followell, 1990) [29].

## 3. Theoretical Background of the FCC

### 3.1. Food Safety and Security

Food safety and food security belong to different scientific disciplines. Food safety from the point of view of natural sciences, medicine, food technology and commodity sciences describes various aspects of health, probability of illness, poisoning or injury as a result of consuming particular food [30,31,32]. Literature offers various definitions of food safety, distinguished as broad or narrow. In the narrow sense, food safety can be defined as the opposite of food risk, i.e., as the probability of not contracting a disease as a consequence of consuming certain food. Safety value for consumers is divided into three sources of health risk: microbiological (presence of pathogen microorganism) [33,34,35], chemical (naturally occurring in plants, nutrients, contaminants present in the environment as residues of harmful substances, additives allowed in food but exceeding certain concentration limit, residues of pesticides, herbicides, traces of chemicals migrating from packaging, residues of drugs, antibiotics and medicines, residues of detergents and disinfection agents, etc.) and physical (as appearance of external and internal materials of various origin, e.g., splinter, file dust, stones, straw). In their recent report on food safety, Kamboj et al. state that approx. 39 percent of foodborne illness cases occur due to pathogen cross-contamination of food during transport, especially during long-distance transportation, while 50 percent is associated with improper storage or reheating [36]. Similar conclusions were highlighted by Pigłowski [37].

Food security from the perspective of economics and social sciences describes confidence in the food production system, supply chain management, availability, continuity and sufficiency for the consumer and industry now and in future. Food security, as defined by the Food and Agriculture Organization [38] “… occurs when all people, all of the time, have physical, social and economic access to sufficient, safe and nutritious food that meets their dietary needs and food preferences for an active and healthy life …”.

The issues connecting food safety and security, as nonseparable parts of the sustainable food system, were discussed in previous research, e.g., [3,39] and FAO [38]. Both food safety and food security significantly contribute to the total quality of food. Researchers, practitioners and consumers understand food quality attributes mainly as: safety, security, sensory properties, convenience of use, functionality and nutritive properties.

The description, normalization and control of food safety and quality has been formalized by various institutions and organizations, such as the Codex Alimentarius (2003) [30], governmental institutions, e.g., EFSA—European Food Safety Authority, Rapid Alert System for Food and Feed (RASFF, 2020) [40]; US Food and Drug Administration and nongovernmental organizations (see Nakat and Bou-Mitri, 2021) [41] as well as specialized intergovernmental institutions (e.g., FAO, WHO, ISO). Most frequently notified hazards in food originating from European Union were: pathogenic microorganisms and microbial contaminants. Currently, “the increase in the number of alert notifications in the RASFF is particularly noticeable in recent years …” [37] mainly due to temperature abuse. Currently, many of these need to be revised due to the COVID-19 pandemic.

### 3.2. Economic Waste and Losses in the Food Chain

**Figure 1:**
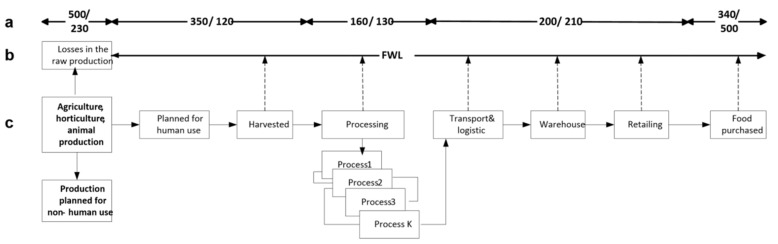
Value of economic food waste and loss (FWL) in the food system. Source: own compilation including data from Hegensholt et al. 2018 [45], where a—exemplary FWL in million tons/billion USD at different stages of production (Hegensholt et al. 2018 [45]); b—continuous FWL process; c—production stages.

There are numerous definitions of the food system (FS) in literature, e.g., [42,43]. One very comprehensive definition states that “a food system gathers all the elements (environment, people, inputs, processes, infrastructures, institutions, etc.) and activities that relate to the production, processing, distribution, preparation and consumption of food, and the outputs of these activities, including socioeconomic and environmental outcomes” [44]. In other words, FS includes all steps from raw production up to the food consumed and all FWL occurring between the source of food up to the consumers’ table (see Figure 1).

Primary agricultural, horticultural and animal production (surplus food) can be divided into (1) intended for human use as food and (2) planned for nonhuman, agricultural and industrial use (e.g., fuels, medicines, animal feed) (see Figure 1). The first components enter into a chain of various processes, which lead to production of food obtained by the consumer. As shown in Figure 1, the food chain from farm to consumer table has a very complicated structure and is linked by many processes, technologies, procedures, standards, norms, etc. Along the processing chain, food waste (FW defined as inedible food) and food losses (FL defined “as food appropriate for human consumption being discarded or left to spoil—regardless of the cause”) occur (HLPE, 2014) [44]. The dashed arrows show the directions of food waste and losses from the field to consumer’s table. The upper arrow line (a) indicates exemplary waste and losses expressed in units of mass (million tonnes) and USD bln respectively (Hegensholt et al. 2018) [45]. It is well-documented, that “about one third of the food produced globally is wasted along the food chain, representing a burden for the environment and an inefficiency for the food system” [46]. Similar data for FW and FL are offered by [45,47] and many others. The upper part of Figure 1 visualizes the magnitude of FW and FL at various stages of the food system (in weight and in monetary value). Lack of technical knowledge and competences, conscious disregard for procedures and rules, lack of human capital, etc. all lead to spoilage of food at different stages. Finally, consumers are wasting a lot of food in their homes, as well, due to overbuying or bad storage (e.g., [19,48]). At the same time, food availability in many countries is limited for economic reasons

From Figure 1, for example, one can read that food processing losses are 160 million tons and 130 billion USD. Unfortunately, it has to be stressed that huge losses occur at the end of the chain, i.e., in consumers’ homes: 340 million tons or 500 billion USD.

To avoid FWL in distribution activities along the food system (FS), it is imperative to use appropriate storage and transport conditions. Poor practices, technical and technological limitations, labor or financial restrictions, lack of proper infrastructure for transportation and storage were identified in literature as major causes of food losses (HLPE 2014 [44], FAO 2021 [49], Gustavsson et al. 2011 [50], Parfitt et al. 2010 [51]). Good storage conditions in which light, moisture, oxygen level, hygiene and sanitation (due to COVID-19 pandemic) and temperature can be controlled and can help reduce FL of perishable products to a large extent (e.g., Martinez et al. 2014) [52]. To decrease FWL in transportation, it is necessary to control and maintain the physical parameters (e.g., temperature, humidity) between the upper and the lower levels in trucks, aircrafts and ships, especially those moving vegetables, fruits or crops (e.g., rice or soy) between continents or distant countries. Perishing of fresh-cut products is accelerated by poor temperature and packaging management (e.g., [53,54]). Even in developed countries, with favorable packaging and temperature management conditions, the number of fresh-cut products perishing is high (HLPE, 2014) [44]. Some authors also highlight the importance of road quality [52,55] and the transporters’ cleanliness and hygienic conditions, which seems obvious but is not always the case [52]. Along the food chain, approximately 15 percent of food losses by weight and value derive from transport, warehouse and retailing activities. However, for a variety of reasons, temperature abuse and improper handling in transport seems to dominate [45,56].

### 3.3. Food Cold Chain

According to Ndraha et al. (2018) [56] the cold chain “is an uninterrupted temperature controlled transport and storage system of refrigerated goods between upstream suppliers and consumers, designated to maintain the quality and safety of food products”. Similar definitions were given by other authors, e.g., [57]. This definition covers all elements of Figure 1, but it is very difficult to fulfill in reality, since the cold chain between primary producers and the final consumer is frequently interrupted during the transport operations. This opinion is shared by Mercier et al. (2017) [58], who states that “the temperature along the cold chain frequently increases above the desired limit, creating food waste and endangering food safety”. These authors highlight groups of operations along the food chain where appropriate temperature might not be observed, namely: precooling, land transportation, display at the retailer and finally transportation and storage by consumers. As the food chain, from primary resources to final products for the consumer, is a multistep process, some operations, transportation specifically, take place multiple times.

However, the relevant literature [59,60] suggests that proper temperature is kept in the manufacturer’s facilities and responsibility lies with them. In fact, a variety of international standards (e.g., ISO 9001, ISO 22000, HACCP) and agencies (e.g., EFSA in the European Union or FDA in the United States) are very rigorous in this aspect. This is not the case in food transportation. In general, there are four temperature categories for food in transport: deep frozen below −18 °C, cold-chilled around 0 °C, medium-chilled at 5–8 °C and finally exotic chilled between 10–15 °C [61]. It is clear that “preservation of quality and safety of fresh food is closely related to the exposure of these products to the optimal temperature” [57].

**Figure 2:**
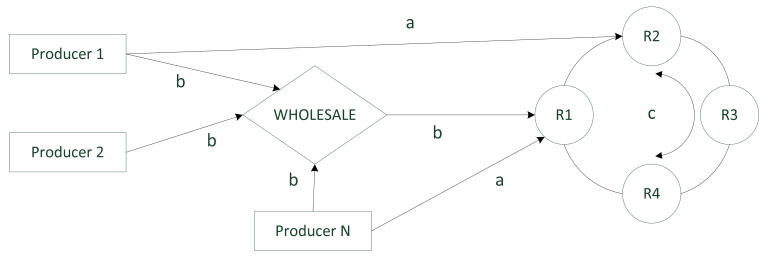
Different modes of transport from producer to retailers (R—retailer, a—direct route, b—chain route, c—circle route).

Land transportation creates problems for temperature maintenance, mostly at the beginning and/or end of the process, while loading or unloading goods. During these activities, temperature might increase even by more than 10 °C [62]. Rising temperatures act to increase viral activity on the surface of, e.g., packaging. Comparable effects were observed in air, railway or sea transportation. The problems may be more severe when a combination of different means of transport (direct, chain or circular route) is used (Figure 2). This figure depicts only a fragment of the FCC between producers and retailers with or without the participation of wholesale.

### 3.4. Temperature Monitoring

#### 3.4.1. Goal and Tools

Monitoring is a systematic process of collecting, analyzing and using information on food’s physical parameters such as temperature, relative humidity and other. However, monitoring is a passive activity, connected with an actual case of time and place. The next shipment will proceed in different external conditions, which are not under control and command of the carrier (e.g., weather, time, destination, distance, etc.). Monitoring in and of itself cannot prevent future issues, nor can it provide an in-depth understanding of the causes of issues in the past. It can only register issues when they arise. The extensive evidence on the impact of temperature on food quality and FWL along the food cold chain calls for radical action throughout all transportation stages.

Based on literature review, the following five key issues need to be considered:

1. Improvement of time–temperature measurement protocols supported by cheap, flexible devices equipped with 3–5 G technology, virtualization and supported by (combined with) SPC;
2. Monitoring, tracking and tracing food product temperatures along the FCC;
3. Application of the results of (1) and (2) in a cause–effect analysis of actual failures in order to understand the transportation process capability in the long-term (period);
4. Elaborating mathematical models of temperature distribution inside containers, trucks, storerooms, etc. for the most important and perishable food;
5. Applying augmented reality tools along the FCC (e.g., Porter, 2019) [63].

To solve the problems of temperature abuse at a low cost, tools and procedures need to be applied in order to achieve an effective working system tracing the temperature during transport. Further on, we describe some already available solutions relating to points 1–3 and their limitations.

One of the pioneering solutions, called TTI (time–temperature integrators/indicators), relies on kinetics of irreversible processes in food (of microbiological, enzymatic and/or photochemical nature) leading to changes of device color (e.g., [64,65,66]). TTI solutions monitor, record and translate the overall effect of temperature history on food quality in the FCC and inform the manufacturer, retailer and consumer of the state of these properties at a given moment. TTI technology is still in use but in declining numbers because the information it provides is bound to the sensor.

Newer solutions are complementary with the paradigm of the Internet of Things (IoT) (e.g., [67,68,69]). We observe a radical evolution of the current Internet into a network of interconnected “devices”, collecting data and information from a vast variety of sensors. The future applicability of IoT depends on the ability to uniquely identify and control these objects (things) among billions of elements in force. Today, Internet standards are able to utilize data to provide services such as information transfer, monitoring, control logistics and predictions [70,71]. Both teams conclude that IoT in agriculture and food production is still in the early stage of development. A current review of the security of IoT was recently published, showing that many improvements are yet to be implemented [72,73]. However, at present, the IoT seems to offer the most cost-effective technical solutions for temperature monitoring.

The building elements of architecture for FCC virtualization by the IoT are:

(A) Hardware (sensors NFC—near-field communication recording temperature, humidity, carbon dioxide, light, etc.); Radio Frequency Identification (RFID); motivator for action (actuator); communication technologies, e.g., Wi-Fi, Bluetooth, Zigbee; energy sources, e.g., solar panels, batteries).
(B) Middleware (a network to transmit and convert data from sensors into protocols, data storage device).
(C) Tools for innovative, easily understandable data visualization and interpretation designed for different applications [60,72,73,74].

#### 3.4.2. Technology

Food storage technology has been changing for thousands of years and is culturally diverse. Food science is evolving using advances from other sciences (e.g., biology, chemistry, physics, agricultural science, engineering). The current IT revolution allows for more precise control of production processes in the food chain and better flow of information. It can also warn managers of processing, storage and distribution processes and of worrying trends that could lead to a crisis (i.e., exceeding tolerance limits). In this situation, the use of modern methods of measuring relevant physical, chemical and microbiological parameters, which are immediately analyzed and send an alert signal via IoT, should be advocated.

Sensors measure temperature or other parameters (e.g., humidity, carbon dioxide, light) of food in a container or vehicle frequently and regularly send the results to an RFID responder. Their task is to signal the value or exceedance of predetermined parameters. RFID technology “is a major breakthrough in the embedded communication paradigm, which enables design of microchips for wireless data communication” (Jayavardhana et al. 2013) [67]. They are now more accurate, easy to use and cheaper [75,76].

This is a result of the maturity and versatile low-cost portfolios of devices, network development and strong support from the business community. The cloud computing market has tripled recently, and only in the first quarter of 2019 it reached 24 billion USD. Especially near-field communication (NFC) [77], wireless sensors network (WSN) or wireless sensor and actuator networks (WSAN) “are the atomic components that will link the real world with the digital one” [78]. An RFID microchip automatically identifies NFC sensors monitoring, e.g., temperature, humidity and air circulation and serves as an electronic barcode [79]. The commercial applicability of the above-presented technology grows due to the replacement of semipassive labels by labels integrated with passive sensors (2G-RFID) [80], which are easier and less costly to use and “provide an environment that registers the conditions and individual characteristics of each food product on its label with a signed mobile code” [81].

Currently, passive sensors (2G-RFID, 50,000 records, without own energy source) are leaders on the market. They are efficient and cheap. They have the following technical performance: the temperature range from −30 °C to +70 °C, a time interval from 2 s to 2 h, the scale of 0.1 °C, precision of 0.2–0.4 °C and the memory capacity up to 50,000 measurements. The NFC standard is used to transfer data to smartphones and other mobile device. These can be connected to a GPS tracking and tracing system and record the history of geographical positions of cargo in transport. For instance, if we know the time–temperature–position history of food from its harvesting or processing time to its storage at the distribution center, then we can avoid shipping food that has been subjected to temperature abuses to a retailer located far away [82]. An NFC Data Logger also enables full temperature traceability during food shipment in isothermal packages or insulated trucks. Temperature readings are transmitted to the “cloud” and stored there. The driver, operator or freight receiving partner can access the historical records on a smartphone in a graphical or numerical form. In addition, some NFC Data Loggers have green and red diodes, showing if the temperature was abused or not, and even send an alert signal to receiving staff.

#### 3.4.3. Current Limitations

The application of technologies to monitor temperature in real time is presently an accessible strategy to reduce food waste and to improve the monitoring of cargo quality [83]. According to David Tanner (2016) [84], RFID technology is also promising for predicting the future value of physical parameters influencing the quality of food and the reduction of waste economic cost. Undoubtedly, the progress in this field will help decrease economic implications of FWL and, indirectly, their environmental strain. However, many scholars [56,85,86] present ample evidence on temperature abuse despite the technical and technological development, especially in transport.

**Figure 3:**
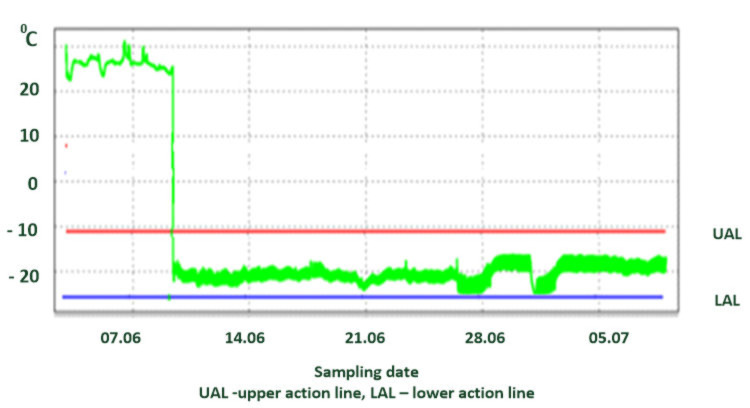
Exemplary temperature fluctuations in a deep freezer with frequent up and down incidents. Source: own experimental data.

One of crucial limitations of current technological solutions in this context can be found in the fact that in a typical monitoring process a significant part of the information about the causes of process variability is hidden or lost. Unfortunately, unlike food processing companies, transport companies pay little attention to identifying causes and preventing improper temperature of the transported cargo. For most carriers, the use of NFC is perhaps a necessity forced by retailers but not an interesting or important solution to investigate and develop further. Operators are interested in an alarm signal about exceeding the temperature limit but not in understanding the complex causes of failures in the transport process. In addition, temperature is typically recorded very frequently, with singular incidents blurring into a larger segment, making it difficult for the user to draw conclusions from the readings [87,88]. This phenomenon is demonstrated in Figure 3.

Scientific literature offering viable solutions to these problems is scarce. Researchers most often present model experiments conducted under laboratory conditions, using low-weight trials (e.g., [88,89]). Some investigated the temperature variation inside a product. Carullo with coworkers (2009) [90] used specially designed measuring nodes that were inserted into products. These nodes communicate with a base station, which collects and retrieves data. A similar experiment was conducted by Lorentzen et al. (2020) [89], De Frias et al. (2020) [91], Abad et al. (2009) [88] and others. Some authors (e.g., Emenike et al. 2016) [92] describe a combination of real-time monitoring of CC ambient parameters with the modeling of future load temperatures in the loading space using artificial neural networks. Another team of researchers modeled temperature within a confined space using multipoint sensors distributed inside a cold chain truck [93]. The Taguchi method (1986) [28] was used to choose the optimal distribution of the load by Mercier et al. 2017 [58]. Another stream of research deals with the estimation of temperature in refrigerated containers [94] as well as in pallets and crates [95]. All these solutions are, however, either complicated or resource-intensive (manpower, time, costs, skilled technicians), hence their limited applicability.

## 4. Results and Discussion

From the mid-1980s onward, the TQM rules and the ISO 9000 series standards were introduced to production management. Quality management in enterprises made the application of statistical tools, including control cards, necessary. Statistical process control (SPC) is now a widely applied tool in control and improvement processes in manufacturing, but research on the successful application of SPC in the food processing industry has been published only recently (Lim et al. 2017) [60]. Information on SPC use in food transportation is rather scare.

### 4.1. SPC in the Food Sector

The food chain from the farm to consumer table is known for its variability and difficulty to control. The problems start with nonhomogeneity of raw materials (agricultural, horticultural, vegetable, animal origin), diversity of recipes and processing methods, seasonality effects, varying weather conditions and many other independent factors. All of these factors influence the quality of the final product. Some other factors, e.g., of chemical origin (fertilizers, pesticides), microorganisms and especially the COVID-19 virus or of physical origin (temperature, humidity, radiation) also affect the safety of foodstuffs. The growing importance of quality, consumer expectations, sanitary regulations, market competition, etc., in the food sector makes the use of SPC tools a necessity (e.g., [96,97]). Several reviews of SPC application in the food industry have been published lately [60,98]. These reviews conclude that food companies implementing SPC have attained significant benefits in terms of reducing process variations, compliance with food regulations, productivity increase, boosting customer confidence and trust, improving continuous process control and process improvement activities. Among the identified limitations, the following issues were the most significant: the lack of statistical thinking, the complexity of SPC tools and the large numbers of variables. The two aforementioned papers, however, do not refer to the statistical backgrounds of SPC. These problems inspired Lim and Anthony to prepare a guide for practitioners and managers in the food industry [99].

### 4.2. Management of Temperature in the FCC Augmented with SPC

Despite its aforementioned limitations, we found that the application of SPC in temperature management in the FCC offers a way to identify and mitigate many of the typical temperature abuse problems.

**Figure 4:**
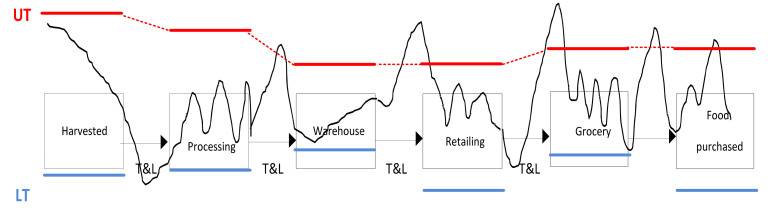
Exemplary lower/upper temperature limit line at different FCC stages. Typical profile of temperature changes in the FFC from rural production to consumer table. Source: based on Mercier et al. (2017) [58]. Abbreviations: UT—upper temperature, LT—lower tolerance temperature, T&L—transport and logistics.

In their review, Mercier et al. [58] highlight the following operations along the food chain in which specific problems regarding the FCC may occur: precooling, land operations during transportation (loading and unloading), transportation (some transport operations may be multiple due to the organization of the supply chain), display at retailers, and finally, transportation and storage by consumers (see Figure 4). 

Of course, the lower and upper temperature limits can vary at each stage of the FCC within the life cycle of the product and are bound to be different for each product. For example, baked goods made from grain will have a different tolerance range as row materials; flour mix; prebaked frozen half-product from an industrial bakery; and, after being baked ready to the in-store bakery, as a final ready-to-serve bread product on display.

Special attention was given to failures at times of loading trucks, causing temperature variations at the center and near the surface of the roof, walls or corners [100]. Similar problems may apply in large storerooms as well. Many problems occur in supermarkets, where various categories of products (dairy, eggs, fruit, vegetables, fresh fruit, meat, frozen food) are displayed at wrong temperatures [58] and in wrong humidity conditions, leading to deterioration of quality and ultimately causing waste.

The technical condition of the means of transport and refrigeration equipment is an essential factor for temperature stability. The homogeneity of the load category facilitates loading and unloading and maintaining the desired temperature [58,82], reducing the time of operation for loading and unloading. Ambient and other weather factors are significant and affect the temperature of the interior and the duration of transport. Skills, experience, honesty, knowledge, stress resistance, drivers’ integrity and conduct in accordance with the principles of good practice in business are very important as well [101].

**Figure 5:**
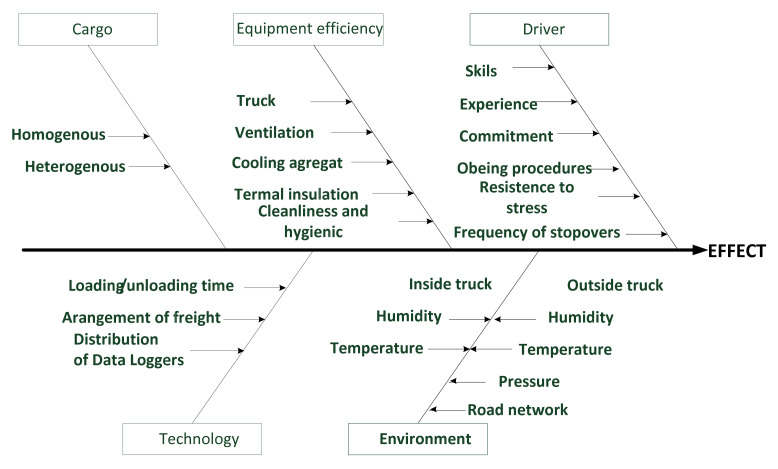
Cause-and-effect analysis in the transportation process on cargo temperature. Source: Figure was created as a result of a brainstorming session with CEOs of a large trade and transportation chains, retailers, quality and transportation managers and scholars (see details mentioned in the last paragraph in Section 2.2. Experimental part. It is our own compilation.

To further dissect the problem, we interviewed managers and professionals using the cause-and-effect analysis method, the results of which, shown in Figure 5, are in agreement with the literature data presented above.

However, after much experience with COVID-19, some of the factors mentioned above need to be given special attention and developed. One of the most important characteristics of a pandemic is driver’s responsibility and adherence to numerous personal hygiene rules. These include: hand hygiene practices, sanitization and disinfection, avoiding close contacts and use of protective equipment. The driver should avoid contact with cargo packaging and on-board equipment. Furthermore, he must take care of the microbiological cleanliness and disinfection of the vehicle by using professional disinfectants or, where possible, hot water [102]. Employees’ body temperature measurements and their vigilance, responsibility and hygiene of the workplace, in this case, the means of transport, are the highest rated, according to a study of 825 processing plants in 16 countries (Djekić I. et al. 2021) [103]. This study also found that retailers are the food chain link mostly affected by pandemic. The weakest point was the lack of emergency plans associated with pandemics. It follows that competency-enhancing training is desirable among those employed in the various links of the FCC.

It has been observed repeatedly that cold chain transportation in the frozen food industry may have caused a recurrence of COVID-19 cases in destination [104]. Chi Y. et al. (2021) analyses the factors of SARS-CoV-2 survival and transmission in different places and environments, especially the characteristics of low temperatures and object surfaces [105]. It was found that SARS-CoV-2 could survive on surfaces of cold and moist objects in the cold chain for more than 3 weeks, potentially causing COVID-19 transmission. To avoid such situations in the future, ideas are being considered to create a new generation of safe cold chain more oriented toward transport than quality and that which merely concerns the very basic part of food safety (chemical contamination and bacterial poisoning).

In Galanakis’s opinion [106], there is undoubtedly a need to avoid “business as usual” practices, to think out of the box and to accelerate efforts to develop sustainable and modern food systems, e.g., to reduce the cost of aseptic lab-grown meat, reduce the cost of food waste recovery and reutilization in the food chain and develop new and large food supply chains based on insects’ and microalgae proteins”.

Recent studies reported some improvements of carriers’ and retailers’ concern regarding temperature control evaluated risk in the FCC using the FMEA (failure mode and effect analysis) approach and developed improvement strategies [107,108,109]. They found “namely long cargo handling time, temperature abuse and product damage” as the most important causes. However, the methods and technologies used in the FCC are prone to failures due to a sudden change of environmental and operational conditions. For example, many problems occurred in 2018 and 2019 in the European countries, caused by an extremely hot summer. In food supermarkets and smaller grocery stores, many deep freezers and refrigerated display cases malfunctioned, resulting in significant FL and problems with insurance companies. Another such example is COVID-19. The rise of the global pandemic creates many challenges for the food system, e.g., problems with cross-border transport and a shortage of trained staff, thus creating additional disruptions in the transportation network [41]. These recent adversities highlight the importance of monitoring and controlling of the physical parameters of food during transport and the surrounding atmosphere (e.g., humidity, ethylene concentration, ozone concentration).

The case of coronavirus demonstrates that such a concept needs to be considered in haste, and perhaps intensively researched (Zhang X-R, 2021) [110]. The author presented two sides of the problem: “On the one hand, super cold chain will certainly promote the development of science, such as logistics, thermo-science, energy engineering, biology, and so on. On the other hand, the development of regulation cannot be ignored, including perfection of system, innovation of technology, construction of infrastructure, standardization, train of talents, and so on” [111].

**Figure 6:**
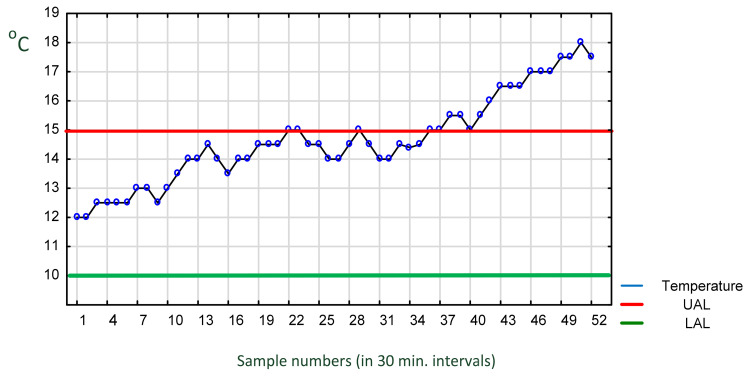
Exemplary record of temperature inside a truck with steadily increasing temperature crossing the upper tolerance. Source: own data collected in 30 min intervals (samples 1–22 daytime driving, 23–37 overnight stop, 37–52 continued driving).

**Figure 7:**
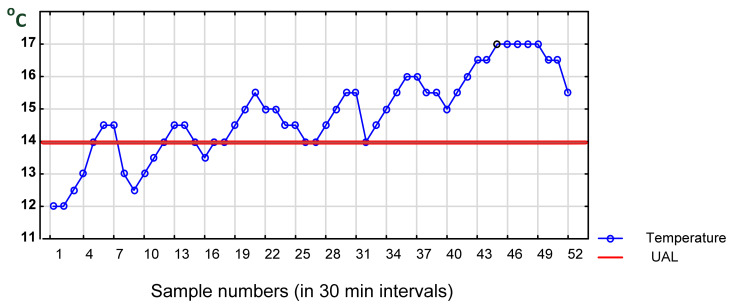
Temperature trend due to repeated opening/leaving them open and closing of a refrigerated truck. Source: own data collected in 30 min. intervals.

These viruses, being RNA viruses, are found to be susceptible to ozone. Ozone being an unstable molecule can break up into its split products, namely reactive oxygen species and ozonides, creating a toxic environment for these viruses [112,113,114]. Some authors (Mazur-Panasiuk et al. 2021) [115] are skeptical about the effectiveness of this method for food transport. Unwanted fluctuations in temperature as well as other parameters (e.g., humidity and air composition) can be mitigated by combining appropriate simple statistical tools with new technology of measuring temperature and/or other physical variables and recording them, as the latter is described below. This may be achieved with the aid of a simple chart (Figure 6 and Figure 7). The following examples demonstrate the use of SPC-augmented technology in selected situations. 

#### 4.2.1. Truck Transport with Frequent Stops

As mentioned previously, in food transport, the following variables are critical for quality: temperature, air composition, microbiological purity and, frequently, humidity of cargo. However, the content of these variables depends on the location of the cargo inside the truck and/or container. The distribution of temperature (or other variables) inside a truck depends on the heterogeneity of the air flow from a fan system and the cargo layout inside the trailer (Mercier et al. 2017 [58]). An important piece of information is also the geographical position (GPS) of the truck. The same location for a longer period of time might indicate technical failure, stopover with open doors, stopover for rest or one with the cooling installation turned off. In this case, the temperature will increase steadily and might cross the upper tolerance limit. Figure 6 presents an example of a graph demonstrating such a situation. The graph, called a “run chart” is a graphical representation of observed data in a time sequence.

Additionally, the lower and upper temperature tolerance lines allowed for the food being transported are shown. After a relatively small variation of temperature between the lower and upper temperature range, we observe a steady upward–downward trend near the upper tolerance limit during the night. Then the temperature rises. This plot of the graph indicates complete lack of temperature control and response on the part of the driver.

#### 4.2.2. Frequent Reopening of a Refrigerated Truck

In the case of a refrigerated truck, food storehouse or a supermarket, for example, temperature measurements are recorded in cargo space, refrigerators, deep freezers and on shelves at time intervals. The pairs of data (temperature and time) for each device, e.g., NFC Data Loggers, can be stored at desired time intervals in the cloud and later retrieved and analyzed using SPC. Results of those measurements and their analysis can be displayed on a smartphone screen of the driver or a warehouseman in a storehouse or a supermarket, triggering an alert and forcing corrective action. In addition, the alert signal could be sent to a responsible manager. If necessary, data can be printed and serve as firm evidence.

Figure 7 demonstrates the temperature fluctuation trend caused by frequent opening of doors, time of leaving them open, unloading and closing the doors of a refrigerated truck or cabinet. Such situation typically occurs in transport when doors to a car, case or icebox are frequently opened by transport operators [91]. This activity depends on the day of the week, hour of the day and the season as well as the outside temperature. It is more reasonable to combine individual readings every few minutes, forming samples in different places of a unit. Samples show both the mean temperature value and range. Moreover, sampling will help reduce the frequency of alarm incidents. This procedure can be applied especially in cases of changing temperature in refrigerated trucks, refrigerators and cabinets in shops (which are frequently opened and closed by customers). This case will need some on-site experiments to optimize the sampling procedure. This procedure will also be helpful for planning the loading of mixed goods when transporting. According to de Frias et al. (2020) [91], “only a handful of studies have evaluated the effect of repeated door openings on product temperature and energy consumption with contrasting reports”. This opinion strengthens our call for optimizing experimental research in the future.

### 4.3. Process Management

The simple graphical presentation of the collected information demonstrated above can also be used to check whether the temperature-management process is “in” or “out” of control. It reflects the process’ variability and detects periods of unusual high or low temperatures and up or down trends present at certain times and in certain phases of the process. It provides information about the violation and disintegration of the cold chain on the basis of individual pieces of data. By monitoring earlier-stage variables (such as duration of a particular leg of a transportation route or necessary night stopover or time gap at a transit point), managers can identify when and in which transport unit the process is “out of control”. This improves understanding of the impact of temperature and time on quality as food is transported, helps increase supply chain visibility and leads to better control of the product quality. However, such activity requires continuous analysis and improvement. Identification of trends will result in improved resource allocation and in mitigation of the identified problems in future operations. For example, the additional information can be used by managers, who will change driver’s instructions or adapt the loading schemes, relying on the accuracy and spread of data from past transport operations.

In order to obtain reliable results, the next step is the analysis of causes for failures, which should be conducted in a set of controlled experiments, executed and analyzed with the application *n* of statistical process control methods.

### 4.4. Process Stability and Accuracy

**Figure 8:**
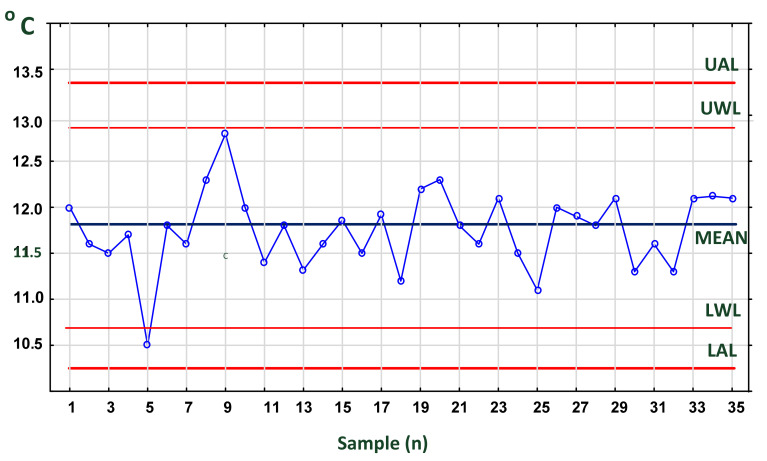
Mean control chart for temperature samples measured in a container at 2 h intervals. Source: own data. Abbreviations: UAL—upper action line, UWL—upper warning line, LWL—lower warning line, LAL—lower action line.

**Figure 9:**
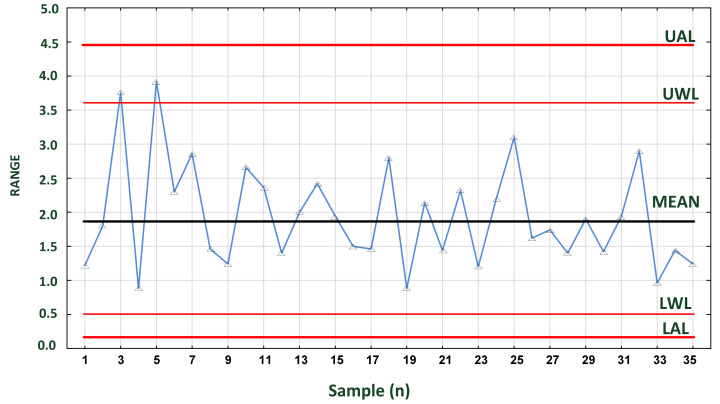
Range control chart for samples of 4 temperatures measured in a container at 2 h intervals. Source: own data. Abbreviations: as in Figure 8.

“To control a process using experimental data, it is necessary to keep a check on current state of accuracy (central tendency) and precision (spread) of the data distribution. This may be achieved with the aid of control charts” [97]. The accuracy of the process is analyzed using mean cards (Figure 8), while its precision is analyzed using range cards (Figure 9). Temperature, humidity and many physical variables are subject to normal distribution (Gauss distribution). Statistical process control is based on simple principles, all of which apply to continuous processes commonly found in food manufacturing, storage, transportation and, finally, distribution. One of them is the law of inevitability or the spread of results, which means lack of stability. Both mentioned authors state that “variability cannot be ignored; it must be recognized and managed”. This type of variation comes from a multitude of outcomes that are “not under full control”. In the FCC, the temperature of cargo depends on many factors (see, e.g., Section 4.2 and Figure 5).

Lack of process stability occurs due to two simultaneous causes: “random” or “assignable”. The random part of variability is explained as the precision of the process. Its statistical measures are: standard deviation—σ, variance—σ^2^ or range.

**Figure 10:**
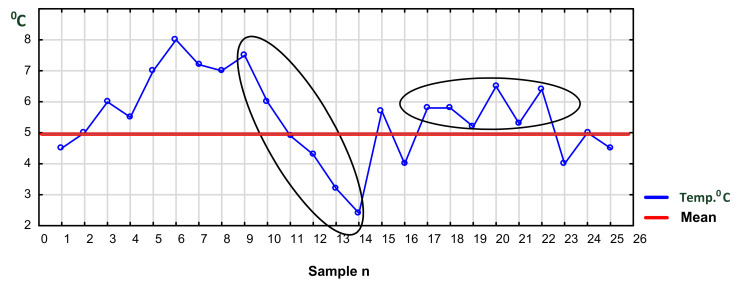
Some specific configurations of 5 or more samples (upward or downward) or five or more points above (or below) a mean line. Source: own data.

Assignable causes occur as part of variation caused by control procedures, inspections, supply system, quality of resources, staff experience and environment, failure of technical resources, etc. Most frequently, they have a strong influence on the total variation. If they occur, the variability of a process is “not under statistical control”. The presence of an assignable cause is signaled by either a point plotting outside the control chart limits or special (characteristic) patterns in the data points (e.g., Figure 10).

It is necessary to find and investigate the nature of assignable causes and observing the results of consecutive transports or in a set of special field experiments. In the next steps, one must eliminate them gradually. We suggest process stabilization methods (for details, see [97]). If only random variations are present in a process, we can call it “under statistical control”. Randomness is caused by small and irregular variations of causes that cannot be anticipated, detected, identified nor eliminated. It is a sum of many small variations inherent in and part of process. Such variation cannot be tracked to root causes.

The second important parameter describing the process is accuracy, which relates to the ability of a process to hit the optimal target value. Its statistical measure is mean, median or mode value of measurements. Only if variation and accuracy are within appropriate range is the process under statistical control. Both parameters characterize normal distribution of continuous variables for a set of temperature measurements and are commonly used to examine process stability over time. This is possible with the mean chart (Figure 8) and range chart (Figure 9) applied simultaneously, if possible.

When the process is under statistical control, 99.7% of the samples will lay between the lines marking the upper UAL and lower LAL action lines. In other words, only three temperature readings among 1000 will lay beyond. It is recommended that lower (LT) and upper (UT) tolerance temperatures for a given cargo are, respectively, below or above action lines. In our case, we used exotic chilled food. According to Gustafsson et al., UT equals 15 °C, and LT equals 10 °C [61]. Finally, the probability that a single reading in 1000 will cross UT or LT lines is very small. Ultimately, two or three consecutive readings in the yellow space (between AWL and LWL) or one or two readings in the red one (between UAL and AWL or LWL and LAL), known as “assignable” cause, require inspection of the appliance, freezer, cooling installation, etc. for technical failure and other causes (see Figure 5).

On the mean card, sample **5** exceeded the LWL, and sample 9 came very close to the UWL (Figure 8). In contrast, on the ranges card (Figure 9), samples 3 and 5 exceeded the UWL. This indicates that the temperature was not stable during the initial transport phase. It can be concluded from these data that the process is not stable in the initial period. Subsequent results indicate that the process is stabilizing.

In addition, a run of five or more readings continuously decreasing (or increasing—not shown) or located above or below the mean line indicates a non assignable process (process out of control). Such process needs immediate action. The occurrence probability of such sequence of points is very small (*p* < 0.005) in a process under control.

The difference (UAL − LAL) in mean charts divided by 6 σ is called the capability index:

C_P_ = (UAL − LAL)/6σ

Its value less than 1 means that the random variation is greater than the tolerance band and that the process is incapable. Values greater than 1 mean the opposite, i.e., that the process is under control. However, the higher the value, the stronger the capability and control.

**Figure 11:**
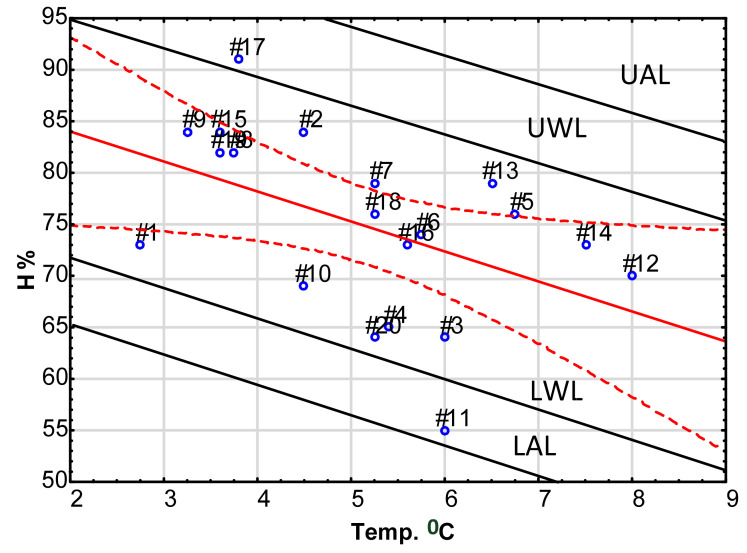
Regression control chart for temperature vs. humidity. Source: own data. Numbers at points # refer to sample number.

The quality of food depends on many factors and must satisfy various standards (see Figure 1). During transportation, inaccurate temperature and other parameters (e.g., humidity, concentration of carbon dioxide, ethylene or other parameters) can severely decrease the quality of food cargo. Monitoring of two or more parameters against temperature on two or more individual charts might mask some unwanted effects and delay the corrective action. In such a case, if it is scientifically reasonable (i.e., when both parameters are independent), one can make use of the linear regression control card, e.g., for temperature and humidity or humidity and ethylene concentration [96,116,117]. This possibility is not frequently used now, even in the food processing industry. However, its potential is large, albeit unexploited. An exemplary regression card is presented in Figure 11 for temperature and humidity inside a container.

The construction of a regression control card differs from that of mean or range control cards. Each axis is scaled in appropriate units (in temperature and relative humidity). Experimental points for a certain time (from 1 to *n*) are presented as a points on the area of the graph. The regression line is equivalent to the central line. In addition, confidence lines (95%) are shown. Above and below the regression line, warning and action lines are indicated.

## 5. Conclusions

This paper has argued that statistical process control of temperature (and other parameters) can play a major role in meeting specific challenges of the food cold chain. Good quality and safety of perishable food can be destroyed by inadequate transport, processing, storage and maintenance along the food chain from the field to the consumers’ table. At each stage of the FCC, various factors and conditions, expected as well as unforeseen, may occur and decrease the quality of food, spoil it or turn it into waste. The abuse of temperature is widely considered as one of the most important parameters decreasing the quality of perishable food along the whole food chain, i.e., at the beginning of the chain (primary production, handling, and storage) and in transport, distribution, retail and, ultimately, shortly before consumption. In addition, it increases economic losses.

According to current data, approx. 1.3 billion metric tons of food are wasted worldwide every year [45]. This means a waste of various resources, e.g., 1.4 billion hectares of agricultural land (approximately 30% of total), or 250 km^3^ of drinking water (approximately 20% of freshwater consumed) or emission of 4.4 giga metric tons of CO_2_ (ibidem). At the same time, access to food in many countries is limited for economic reasons. Each link along the chain requires appropriate temperature to maintain food properties and quality. Along these many stages during production, transportation and storage, however, the temperature distribution is variable, diverse in space and time. It has been extensively documented that temperatures in trucks, palettes, cold stores, refrigerators, storerooms, etc. often abuse reference limits for specific foods.

After an analysis of literature on the FCC we observed the lack of systematic research on temperature changes of food along the entirety of the supply chain. We also found that, despite the maturity of the technical system, only a part of the potential possibilities and information it offers is currently extracted and applied to stabilize the temperature management process in transport. In contrast, some works show that the food processing industry is without large faults, mainly due to strict controls of production processes. SPC tools in the form of control charts and other graphic tools are commonly used in this industry to manage quality. However, food processing covers only a part of the whole FCC.

The Internet of Things presently achieved the capability of retrieval and processing of a vast amount of data, all thanks to the present Internet G4 technology and the emerging G5. The integration with GPS further expands the capabilities of devices in transport processes. Following the analysis of reviews of technologies used to maintain and control temperature in the FCC, a theoretical solution for more comprehensive temperature management was identified and tested. We suggest the use of a technical system, comprised of RFID Data Loggers and sensors detecting specified parameters (e.g., temperature, humidity, concentration of CO_2_ or ethylene and other necessary physical parameters or concentrations) in real time, a wireless sensor network and cloud computing and augmenting this system with methods of SPC.

We documented that:

A gap in the literature was identified in the study of reasons for FWL in transportation versus processing due to temperature overshoot.

A cause-and-effect analysis was performed in the form of management interviews with CEOs of large shipping companies, food wholesalers, retail chains, IoT professionals, NFC Data Loggers manufacturers and researchers. Five groups of factors with significant impact on food temperature during transportation were generated.

It was shown that food carriers using modern temperature recording tools at most react to an alarm signal, but they do not analyze the causes. Cargo temperature varies in transit depending on various causes identified by cause-and-effect analysis (including duration of transport, outside temperature, quality of truck insulation, location inside a truck, number of stopovers and even the driver’s skills and honesty).

Data collected by NFC Loggers can be used more extensively than it is currently for graphical presentation and triggering an alarm only. As presented in this paper, such data can be also used for generating SPC run, mean, range or regression charts. These charts enable detection of assignable causes and random ones for failure in a given part of the FCC. This can greatly improve the knowledge of all processes management along the FCC.

The questions arise: how many and where should the sensors for ambient monitoring be located and how often should the data be recorded. The answers to these questions should result from specially designed and simulated in-field experiments. The problem can be solved in the future with the aid of statistical experimental design (e.g., Box et al. 2005) [118], the Taguchi method (Taguchi G., 1986) [28], or neural networks and other (see e.g., Badia-Melis et al. 2018 [95]. These methods allow researchers to achieve an optimal solution with a reasonably small number of statistically planned experiments.

To tentatively answer this question, we conducted and analyzed several simulated in-field experiments. These were based on statistical scheduling of experiments (e.g., Taguchi, 1986; Box et al. [28,118]), which allow for a reliable solution with an optimal number of experiments.

In this case, it is necessary to conduct research on temperature gradients as a function of time of day, transportation route, number and duration of stopovers, length and frequency of unloading, periods of open doors and others causes identified in our work. The measured data should be used for predicting the cause and moment when temperature might cross upper or lower action limits.

Improving scientific research on methods for measuring and interpreting changes in temperature and other parameters in the CC (e.g., humidity) is desirable for at least three reasons.

1. There is possibility to apply in road or rail transport over long distances, e.g., Spain–Eastern Europe (about 2900 km), China–Europe (about 12,000 km) or sea transport, which lasts several weeks, the use of other SPC tools that were not described in this paper. Here we can mention moving average charts and exponentially weighted moving average charts (EWMA) with reduced frequency of measurements. According to Holt, they allow prediction of future changes of parameters [119] and the ability to react in advance.
2. Efforts are needed to increase familiarity with the essence of SPC tools in transportation activity. Previous experience of more than a century since the introduction of Shewart’s cards indicates that the knowledge of simple statistical methods is the key to success but was difficult to access for workers in various areas of industrial activity. This difficulty became apparent after the introduction of ISO 9000 standards and the HACCP system in the food industry [99]. Now it is time for transport companies. They required employees with strong capital attributes such as integrity, faith and dignity, competence, responsibility and honesty, values that are closely related to motivational instruments for effective work in the driver’s seat and many other workplaces along the FCC. The realization of such intentions requires continuous investigation in addition to knowledge of statistical tools and properties of a constantly mutating virus.
3. It was found that SARS-CoV-2 could survive on different surfaces of cold and moist objects in the cold chain for more than 3 weeks (depending on temperature), potentially causing COVID-19 transmission after freezing [105,120,121]. This applies not only to transport but also to the downstream parts of the food system [122]. Smith et al. documented a significant negative linear effect of temperature on SARS-CoV-2’s R0 and a significant positive effect of population density [123]. Unfortunately, statistical models involving more variables that can significantly influence virus transmission (e.g., humidity, UV intensity) have not yet been proposed. For this reason, temperature is assumed to be the primary environmental predictor. Summer weather cannot be considered a substitute for mitigation policies. Lower autumn and winter temperatures lead to an increase in transmission of COVID-19 in the absence of states policy and changes of human behavior.
